# Identification of a circulating long non-coding RNA signature panel in plasma as a novel biomarker for the detection of acute/early-stage HIV-1 infection

**DOI:** 10.1186/s40364-024-00597-7

**Published:** 2024-06-12

**Authors:** Santanu Biswas, Namrata Nagarajan, Indira Hewlett, Krishnakumar Devadas

**Affiliations:** Laboratory of Molecular Virology, Division of Emerging and Transfusion Transmitted Diseases, Center for Biologics Evaluation and Research, Food and Drug Administration, 10903 New Hampshire Avenue, Silver Spring, MD 20993-0002 USA

**Keywords:** Early HIV-1, Acute HIV-1, ART HIV-1, Plasma lncRNA biomarker and diagnosis

## Abstract

**Background:**

Individuals with acute / early HIV-1 infection are often unaware that they are infected with HIV-1 and may be involved in high-risk behavior leading to transmission of HIV-1. Identifying individuals with acute / early HIV-1 infection is critical to prevent further HIV-1 transmission, as diagnosis can lead to several effective HIV-1 prevention strategies. Identification of disease-stage specific non-viral host biomarkers would be useful as surrogate markers to accurately identify new HIV-1 infections. The goal of this study was to identify a panel of host derived plasma long non-coding RNAs (lncRNAs) that could serve as prognostic and predictive biomarkers to detect early/acute HIV-1 infection.

**Methods:**

A total of 84 lncRNAs were analyzed in sixteen plasma samples from HIV-1 infected individuals and four healthy controls using the lncRNA PCR-array. Twenty-one lncRNAs were selected and validated in 80 plasma samples from HIV-1 infected individuals *[*HIV-1 infected patients in the eclipse stage (*n* = 20), acute stage (*n* = 20), post-seroconversion p31 negative stage (*n* = 20), and post-seroconversion p31 positive stage (*n* = 20) of infection*]* and 20 healthy controls. The validation study results were used to develop a plasma lncRNA panel that was evaluated in the panel test phase to detect early/acute HIV-1 infection in 52 independent samples.

**Results:**

We identified a lncRNA panel (P_model−I_) containing eight lncRNAs (DISC2, H19, IPW, KRASP1, NEAT1, PRINS, WT1-AS and ZFAS1) that could distinguish HIV-1 infection from healthy controls with high AUC 0·990 (95% CI 0.972-1.000), sensitivity (98.75%), and specificity (95%). We also found that P_model−II_ and P_model−III_ demonstrates 100% sensitivity and specificity (AUC 1·00; 95%CI:1·00–1·00) and could distinguish eclipse stage and acute stage of HIV-1 infection from healthy controls respectively. Antiretroviral treatment (ART) cumulatively restored the levels of lncRNAs to healthy controls levels.

**Conclusion:**

lncRNA expression changes significantly in response to HIV-1 infection. Our findings also highlight the potential of using circulating lncRNAs to detect both the eclipse and acute stages of HIV-1 infection, which may help to shorten the window period and facilitate early detection and treatment initiation. Initiating ART treatment at this stage would significantly reduce HIV-1 transmission. The differentially expressed lncRNAs identified in this study could serve as potential prognostic and diagnostic biomarkers of HIV-1 infection, as well as new therapeutic targets.

**Supplementary Information:**

The online version contains supplementary material available at 10.1186/s40364-024-00597-7.

## Introduction

Many diagnostic tests currently in use, are based on the detection of human immunodeficiency virus (HIV)-specific antibodies, and are not unable to detect acute HIV-1 infection (AHI) [1], although a few diagnostic NAT assays are available, there is still a small window immediately after exposure that remains a challenge for early detection. AHI is defined as the period prior to the development of detectable antibodies and can be characterized by Fiebig staging [2–4]. In Fiebig stage I only HIV-1 RNA is detectable, and in Fiebig stage II HIV-1 p24 antigen, a transient viral core protein becomes detectable. AHI is a highly infectious phase where viral load often reaches peak levels, sometimes > 1 × 10^7^ copies/mL [5]. Furthermore, the high viral load and the absence of anti-HIV-1 antibodies contribute disproportionately to HIV-1 transmission [6–9]. In the next phase, immunoglobulin (IgG and IgM) antibodies are expressed which can be detected by 3rd and 4th generation immunoassays [2, 10–12]. In adult infections, it takes approximately 3 weeks, but perhaps as long as 6 months, before seroconversion takes place to generate detectable anti-HIV-1 antibodies [13]. Transmissibility of HIV-1 is high during the acute stage of infection (the first 1–3 weeks of infection) when viral load levels ramp-up in the host and antibodies may not be detected, which underscores the need for reliable testing to inform patients of their HIV-1 status to minimize secondary spread to contacts and partners [14]. Accurate HIV-1 laboratory diagnosis is critical to lowering the likelihood of HIV-1 positive individuals transmitting HIV-1 infection. HIV-1 diagnostic immunoassays and Western blots have recently been systematically characterized in a large cohort of people who initiated ART during acute HIV infection [15]. The use of current ART regimens to treat acute and early HIV-1 infection has demonstrated the benefits of viral suppression, immune function maintenance, and delayed disease progression [16–18]. Additionally, because of viral suppression, early antiretroviral therapy (ART) reduces the size of the viral reservoir, lowers the rate of viral mutation, and improves the management of chronic inflammation [19–21]. The significance of such early HIV-1 detection cannot be overstated since this offers a vital chance for action to stop the widespread dissemination of HIV-1 and reduce further transmission. Thus, there is a need to identify disease-stage specific non-viral host biomarkers as surrogate markers in virally suppressed disease stages, which might be accomplished using a panel of validated host long non-coding RNAs (lncRNAs) elucidated in this report.

LncRNAs are longer than 200 base-pairs and lack protein-coding capability [22]. LncRNAs regulate gene expression and cellular processes by interacting with DNA, RNA, or proteins. Current studies have shown that lncRNAs are capable of regulating viral replication, latency, immune response, drug resistance, and disease progression, making them useful candidates as diagnostic biomarkers, therapeutic targets, or vaccine adjuvants for HIV/AIDS [23]. Trypsteen et al. investigated the expression of lncRNAs at different stages of HIV replication and suggested that lncRNAs may represent targets for controlling HIV replication [24]. Thus far, alterations in plasma or serum lncRNA levels have only been reported in disease conditions related to sepsis [25], tuberculosis infection [26], coronary artery disease (CAD) [27] and cancer [28, 29]. In the context of HIV-1 infection, several lncRNAs have already been demonstrated to play a role during HIV-1 infection in vitro, either by being hijacked by HIV-1 to aid in replication or latency, or as part of the immunological response against the virus [30]. Multiple studies have described the role played by lncRNAs in regulating HIV infection as well as disease progression [24, 31–33]. In 2016, a study indicated that the lncRNA, NRON, was highly expressed in resting CD4 + T lymphocytes [31]. Moreover, Jin et al. reported that the expression of NEAT1 was correlated with CD4 T-cell counts, suggesting that the presence of NEAT1 in plasma maybe be considered as a potential biomarker of HIV-1 infection [33]. Recently, Ma et al. identified several lncRNAs that might play critical roles in HIV-1 replication and immune activation during early HIV infection [34]. However, most of these reports were based on studies using a single candidate lncRNA, a small study population, and no independent validation which limited their scope. Although, research in this field has focused on the intracellular activities of lncRNAs, there is growing interest in the activities of extracellular circulating lncRNAs. This research expands on the concept of circulating microRNAs, which have shown potential as non-invasive biomarkers in a variety of illnesses including HIV-1 infection [35, 36].

In our study, we have aimed to identify circulating lncRNAs that distinguish individuals with HIV-1 infection from those who are free from HIV-1. So far, very few studies have screened and verified circulating lncRNAs in HIV-1 infection [24, 31–33]. All prior studies have reported differential expression of host lncRNAs in PBMCs from HIV-1 infected individuals, but none investigated whether circulating lncRNAs in plasma or serum might identify acute/early-stage infection. The purpose of this work was to examine lncRNA expression patterns from HIV-1 infected patients in the acute/early stage of infection to identify a panel of lncRNA biomarkers that could be useful to detect this stage of infection.

## Materials and methods

### Patient characteristics

Seroconversion panels were purchased from SeraCare Life Sciences, Gaithersburg, MD, USA (eight panels containing 24 HIV-1 positive plasma samples) and ZeptoMetrix Corp., Buffalo, NY, USA (two seroconversion panels containing 10 HIV-1 positive plasma samples). Seventeen HIV-1 positive seroconversion p31 positive samples plasma samples and two AccuSet HIV-1 performance panels containing 38 HIV-1 positive plasma samples were purchased from SeraCare Life Sciences, Gaithersburg, MD, USA. Sixteen HIV-1 positive plasma samples were purchased from The American Red Cross Society, Gaithersburg, MD, USA and 11 HIV-1 positive plasma samples were purchased from Discovery Life Sciences Los Osos, CA USA. All 20 ART-treated HIV-1 positive plasma samples were obtained from Discovery Life Sciences in Los Osos, CA USA.

Samples that matched the following criteria (Table 1) were included in this study. All eclipse stage samples had viral loads that were below detection limits and were antigen antibody non-reactive. These eclipse samples were from initial bleeds from patients who tested positive for HIV-1 in later bleeds. Acute HIV-1 samples have a detectable viral load and are HIV-1 p24 antigen reactive, but non-reactive to anti-HIV-1 antibody. Post-seroconversion p31 negative samples are HIV-1 RNA +, HIV-1 p24 antigen reactive, anti-HIV-1 antibody reactive and are either HIV-1 Western blot reactive without p31 bands or indeterminant. Post-seroconversion p31 positive samples are HIV-1 RNA +, HIV-1 p24 antigen reactive, anti-HIV-1 antibody reactive and HIV-1 Western blot positive with p31 bands. Samples from the early HIV infection phase included eclipse, HIV-1 RNA positive, HIV-1 p24 antigen positive, anti-HIV-1 antibody positive and Western blot indeterminate or positive (with negative p31 band) HIV-1 infected participants receiving ART for at least 12 weeks (standard of care) with undetectable levels of viral RNA or low levels of viral RNAs (< 100 copies/ml) and elevated level of CD4 counts (more than 400 cells/µL). Individuals with hepatitis B and C virus infection were excluded from the study. Based on these parameters, HIV-1 samples were classified as follows:

**Table 1:** Sample categories and their specifications

Specimen Categories	Specifications
Eclipse	HIV-1 RNA below detection limit, antigen, and antibody negative
Acute HIV-1 infection/ pre- seroconversion	RNA positive and Ag positive specimens
Post-seroconversion p31 negative	HIV-1 RNA positive, p24 antigen positive, antibody positive and Western blot indeterminate or positive (except p31 band)
Post-seroconversion p31 positive	HIV-1 RNA positive, p24 antigen positive, antibody positive and Western blot indeterminate or positive (p31 band positive)
Early HIV-1 infection	All eclipse, HIV-1 RNA positive, p24 antigen positive, antibody positive and Western blot indeterminate or positive (except p31 band)
HIV-1 infection	All HIV-1 infected samples
ART HIV-1	HIV-1 infected participants receiving ART for at least 12 weeks with viral RNAs < 100 copies/ml and/or CD4 count > 400 cells/uL

All plasma samples from HIV-1 infected individuals were characterized using commercially available FDA-approved / licensed tests and in-house Research Use Only (RUO) assays

The National Institutes of Health (NIH) Blood bank provided human whole blood drawn from healthy volunteers (36 donors) seronegative for both HIV-1, hepatitis C and hepatitis B in EDTA-K2 tubes (BD Vacutainer, Franklin Lakes, NJ, USA). This protocol was authorized by the NIH ethics committee to use deidentified samples of blood and/or blood products collected under the NIH IRB-approved protocol and permission form (study number: 99-CC-0168, PI: Susan F. Leitman, M.D.). To extract nucleic acid-free plasma, the samples were spun three times at 4 °C (1500 r.p.m. for 30 min, 3000 r.p.m. for 5 min, and 4500 r.p.m. for 5 min) [37]. Plasma samples were then kept at -80 °C until they were analyzed.

### Study design

**Fig. 1:**
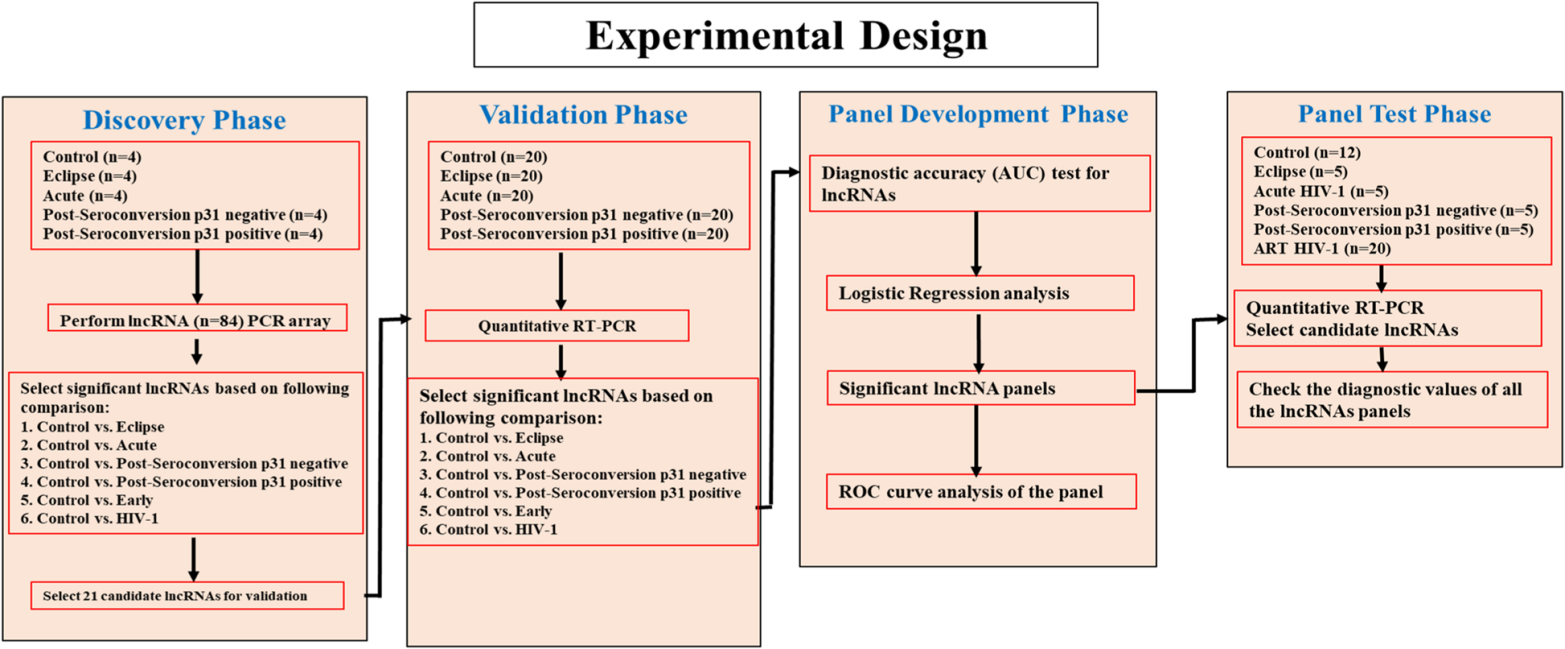
Study Design. Results were produced in two phases using the long noncoding RNA (lncRNA) profiles from 120 plasma samples from 96 HIV-1 infected individuals and 24 healthy uninfected individuals (control). To confirm the candidate lncRNAs discovered in 20 plasma samples using lncRNA PCR arrays, 100 plasma samples were used for quantitative RT-PCR. The logistic regression and ROC curve analyses were performed in the validation panel development stage. The panels were further validated in the panel test phase with 52 additional plasma samples

The experimental plan was divided into four phases (Fig. 1): discovery, validation, panel development, and panel testing. During the discovery phase, samples were screened using the lncRNA PCR Array Human IncFinder (Cat# LAHS-001Z) and promising differentially expressed lncRNAs were identified and chosen for further study. In the validation phase, the selected candidate lncRNAs were further assessed. In the panel development phase, the validated lncRNAs were combined to form a diagnostic panel. During the validation phase, the predictive performance of the lncRNA panels to serve as possible biomarkers were also evaluated. The clinical diagnostic performance of the lncRNA diagnostic panels was examined in the panel test phase with samples whose clinical diagnostic information was blinded to us. Clinical diagnostic information, sensitivity and specificity of the logistic models in distinguishing HIV-1 infection, early HIV-1 infection, eclipse stage of infection, acute infection, post seroconversion p31 negative stage of HIV-1 infection, and p31 positive stage of HIV-1 infection from healthy control participants were estimated after unblinding the test findings.

### Total RNA isolation and reverse transcription

Plasma RNA was isolated as previously described [35]. Circulating lncRNAs were reverse transcribed using the RT^2^ lncRNA PreAmp cDNA synthesis kit according to the manufacturer’s instructions (Cat. # 330,451 Qiagen, USA). This includes an initial genomic DNA elimination step, isothermal cDNA conversion, and a 12 cycle preamplification (RT^2^ PreAmp PCR master mix and RT^2^ lncRNA PreAmp primer mix) to improve detection of low copy number lncRNAs.

### LncRNA PCR array and assay

The pre-amplified cDNA was then used to quantify eighty-four predefined lncRNAs using the RT^2^ lncRNA PCR Array Human lncFinder (LAHS-001Z; Qiagen, USA). The array also contains five primer pairs for housekeeping genes (SNORA73A, RN7SK, RPLP0, B2M, ACTB), One for the detection of human genomic DNA contamination, three for reverse transcription control and three for positive PCR control. RT^2^ SYBR Green ROX qPCR Master Mix (Cat# 330,523, Qiagen, Germantown, USA) was used for real-time PCR reaction on Applied Biosystems ViiA7 instrument (Life Technology, USA). In the validation phase, expression levels of 21 lncRNAs was detected with RT^2^ lncRNA qPCR Assays (Cat#330,701, Qiagen, Germantown, USA) and RT^2^ lncRNA qPCR assay for Human RN7SK (Cat#330,701, GeneGlobe ID - LPH28399A, Qiagen, Germantown, USA) and RPLP0 (Cat#330,701, GeneGlobe ID - GeneGlobe ID - LPH28473A, Qiagen, Germantown, USA) reference genes with RT^2^ SYBR Green ROX qPCR Master Mix (Cat# 330,523, Qiagen, Germantown, USA). The measurements were performed in 96-well plates (ABI ViiA7) in 20 µl of total PCR Reaction Mix volume.

### Differentially expressed host lncRNAs

In the discovery phase, differential expression of host lncRNAs was identified using RT^2^ lncRNA PCR Array Human lncFinder (LAHS-001Z; Qiagen, USA) with total RNA isolated from plasma samples from HIV-1 infected patients. A total of twenty plasma samples from HIV-1 infected patients (four Eclipse, four Acute, four post-seroconversion p31 negative and four p31 positive individuals) and four healthy normal controls were used. This PCR array allows for the analysis of 84 human lncRNAs. The cycle threshold (Ct) values were analyzed using the Qiagen web-based software (http://www.qiagen.com/geneglobe). The data analysis web portal calculates fold change/regulation using ∆∆Ct method, in which ∆Ct is calculated between gene of interest (GOI) and an average of reference genes (HKG), followed by ∆∆ Ct calculations [∆Ct (Test Group)-∆Ct (Control Group)]. Fold Change is then calculated using 2^−∆∆Ct^ formula. To determine whether these candidate lncRNAs could serve as effective biomarkers, we performed several pairwise comparisons (control vs. eclipse, control vs. acute, control vs. post-seroconversion p-31 negative, control vs. post-seroconversion p31 positive, control vs. early HIV-1 and control vs. HIV-1). Twenty-one lncRNAs that were differentially expressed in these comparisons were chosen as potential candidates for further investigation.

We measured the relative amounts of the 21 candidate lncRNAs in 80 plasma samples from HIV-1 infected people (including 20 eclipse, 20 acute, 20 post-seroconversion p31 negative, and 20 post-seroconversion p31 positive) and 20 normal controls during the biomarker validation phase. Plasma lncRNAs from the validation stage were analyzed by quantitative real-time PCR. Currently, the use of one housekeeping gene as an internal control is the widely used method for normalization in gene expression studies. However, in many studies reference genes are chosen randomly and are not always validated for the particular experimental conditions. It is noted that under certain experimental conditions the expression of reference genes was affected [38, 39]. In the discovery stage of our experiments we first tested five well established reference genes (ACTB, B2M, RPLPO, RN7SK and SNORA73A). After analyzing the data we decided to use only two reference genes (RPLPO and RN7SK) in the next stage due to their stable expression throughout the various sample groups (Supplementary Figure S1) compared to the other three reference genes. Relative quantitation was used and the levels of lncRNA in plasma samples were normalized against the reference genes (RN7SK, RPLP0) and the results were presented as ∆Ct in which ∆Ct is calculated between the gene of interest (GOI) and an average of the reference genes (HKG).

### lncRNA panel development and testing

To categorize the different stages of HIV-1 infection, the ∆Ct-value of significant lncRNAs was applied in the logistic regression analysis to construct the lncRNA panel, denoted as P_model−I_ that could differentiate between HIV-1 infection and uninfected controls. The predicted probability of detecting samples from HIV-1 infected individuals by P_model−I_ was calculated as: logit (model-I) = 11.866–0.953*DISC2–1.010*H19 + 0.649*IPW + 0.388*KRASP1 + 0.880*NEAT1–1.220*PRINS – 0.728*WT1-AS + 2.318*ZFAS1. In this equation, the lncRNA symbol was replaced with the ∆Ct value of the lncRNA. If the result of logit [model-I] was greater than or equal to 0.0221, the sample was predicted to be from an HIV-1 infected individual.

In the panel test phase, the clinical diagnostic performance of the lncRNA panel established in the validation phase was further examined with an entirely independent set of 52 plasma samples not previously used. After reviewing the clinical diagnostic data, the logistic models’ sensitivity and specificity in distinguishing HIV-1 infection, early HIV-1 infection, eclipse, acute, post-seroconversion p31 negative, and post-seroconversion p31 positive stages from uninfected healthy individuals were determined. The samples utilized in the validation and panel test phases were both independent of the discovery phase and of one another.

### Statistical analysis

To examine the control of type-II errors during lncRNA screening and validation in plasma samples, a basic power analysis calculation was done. Power calculations for different lncRNAs were performed during the discovery stage using sixteen HIV-1 positive samples and four controls using a 2.0-fold change as the cut-off. During the validation stage, results from eighty HIV-1 positive plasma and twenty normal control persons revealed that each lncRNA had 99% observed power.

PCR array data was analyzed for significance to identify the differentially expressed lncRNAs between HIV-1 infected patients and healthy uninfected individuals. Student t-test was used to examine differences in plasma lncRNA expression levels between HIV-1 infected individuals and healthy uninfected individuals. The area under the ROC curve (AUC) and receiver-operator characteristic (ROC) curves were used to evaluate the effectiveness of the selected lncRNAs as diagnostic tools for detecting HIV-1 infection. The Youden index was used to identify the best cutoff point [40, 41]. A linear combination of lncRNAs was created using logistic regression. All statistical analyses were carried out with data that had been normalized and using the ∆Ct values. SPSS software, version 170 (SPSS Inc., Chicago, IL) and GraphPad Prism 9 (GraphPad Software, Inc., California) were used for statistical analyses. All statistical tests were two-sided, and *p* ≤ 0.05 were considered statistically significant.

## Results

We analyzed 172 plasma samples from HIV-1 infected patients and healthy uninfected individuals (control population) to identify circulating lncRNAs that could serve as biomarkers for detecting early stages of HIV-1 infection (Fig. 1). To identify potential plasma lncRNA biomarkers specific for HIV-1 infection, plasma RNA from four uninfected individuals, and sixteen plasma samples from HIV-1 positive individuals (four each of eclipse stage, acute stage, post-seroconversion p31 negative stage and post-seroconversion p31 positive stage) was pre-amplified, and the expression levels of 84 lncRNAs were profiled using the RT^2^ lncRNA PCR Array Human IncFinder (Cat# LAHS-001Z, Qiagen, USA). We performed several pairwise comparisons in the 20 individual samples tested and identified differentially expressed lncRNAs between the groups (Supplementary Figure S2 and Supplementary Table S1).

**Fig. 2:**
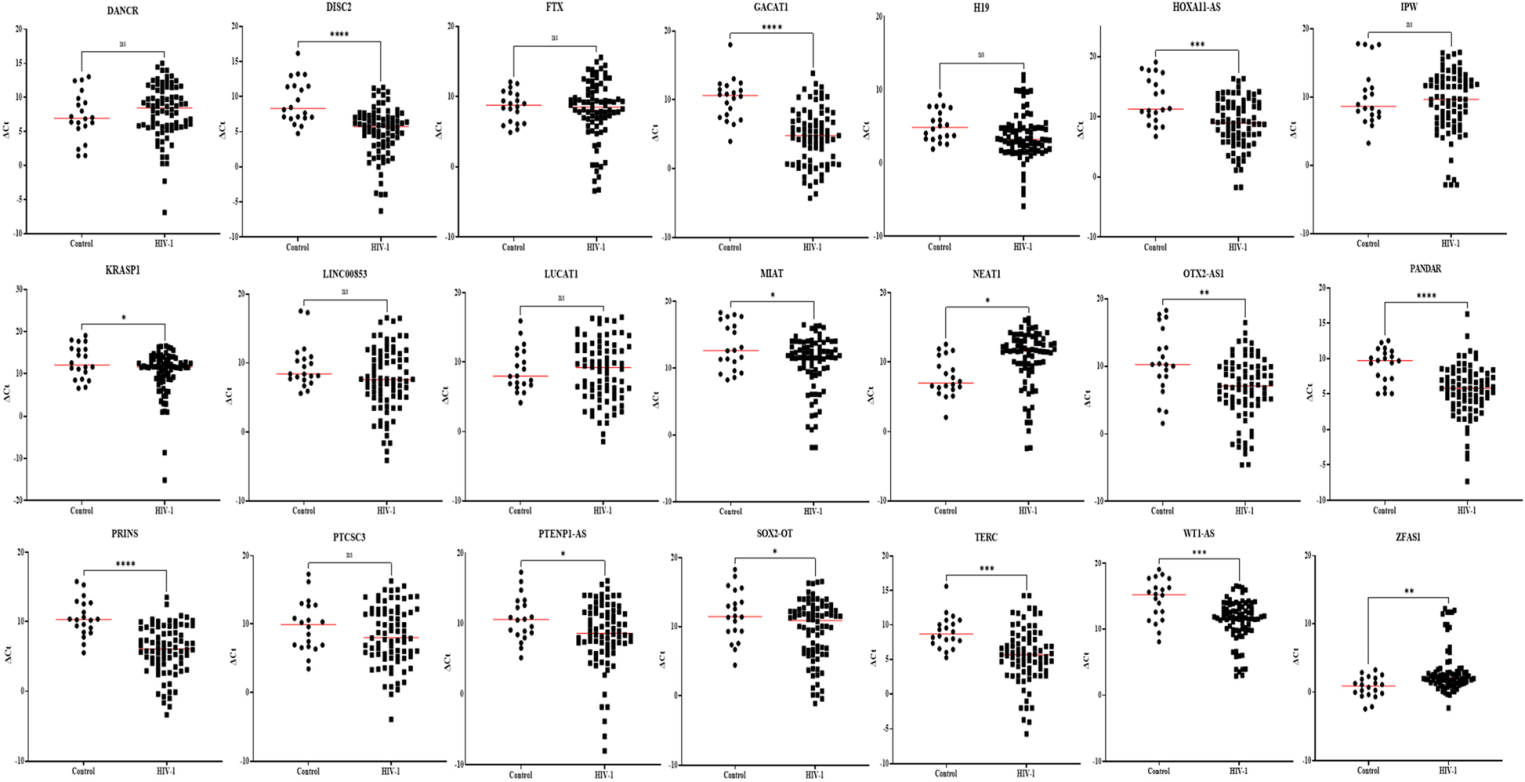
Expression level of plasma lncRNA candidates as HIV-1 infection markers in the validation stage. The level of plasma lncRNAs in 80 plasma samples from HIV-1 infected individuals and 20 plasma samples from uninfected individuals were examined using real-time RT-PCR, △Ct method was used to calculate lncRNA expression, which was normalized with two reference genes (RPLPO and RN7SK). A lower ΔCt value indicates higher lncRNA expression and a higher △Ct value indicates lower lncRNA expression. Comparison of the lncRNA expression profiles in plasma samples from HIV-1 infected individuals and uninfected individuals indicated that the expression of twelve lncRNAs were significantly increased and two lncRNAs were significantly decreased in the plasma from HIV-1 infected individuals. (*p < 0·05; **p < 0·01, ***p < 0·001, ****p < 0·0001 and ns = non-significant as calculated using Student’s t-test)

**Fig. 3:**
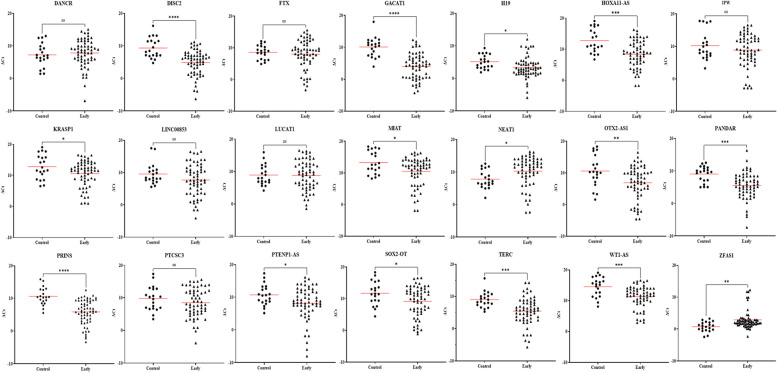
Expression level of plasma lncRNA candidates as biomarkers for early HIV-1 infection in the validation stage. The level of plasma lncRNAs in 60 plasma samples from HIV-1 infected individuals and 20 plasma samples from uninfected individuals were examined using real-time RT-PCR, △Ct method was used to calculate lncRNA expression, which was normalized with two reference genes (RPLPO and RN7SK). A lower ΔCt value indicates higher lncRNA expression and a higher △Ct value indicates lower lncRNA expression Comparison of the lncRNA expression profiles in plasma samples from HIV-1 infected individuals in the early phase of infection with the samples from uninfected individuals indicated that the expression of thirteen lncRNAs were significantly increased and two lncRNAs were significantly decreased in the plasma from HIV-1 infected individuals. (*p < 0·05; **p < 0·01, ***p < 0·001, ****p < 0·0001 and ns = non-significant as calculated using Student’s t-test)

**Fig. 4:**
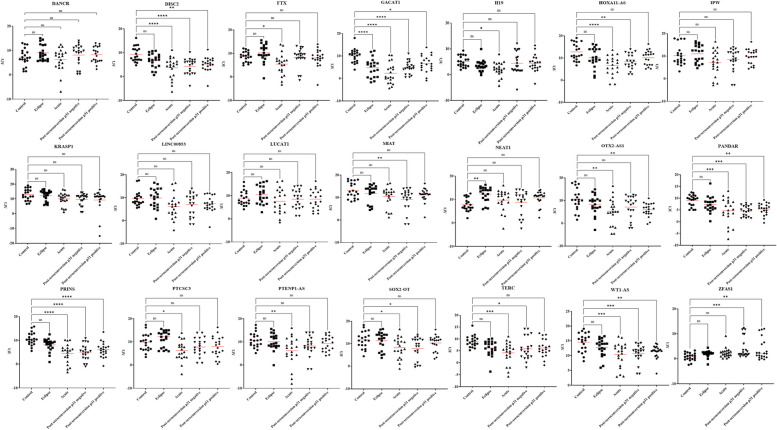
Expression levels of plasma lncRNA candidates in different stages of HIV-1 infection from the validation stage. The level of plasma lncRNAs from HIV-1 infected individuals in eclipse (*n* = 20), acute (*n* = 20), post-seroconversion p31 negative (*n* = 20) and post-seroconversion p31 positive (*n* = 20) stage and 20 plasma samples from uninfected individuals were examined using real-time RT-PCR, △Ct method was used to calculate lncRNA expression, which was normalized with two reference genes (RPLPO and RN7SK). lncRNA expression and a higher △Ct value indicates lower lncRNA expression Two lncRNAs (one down regulated and one up regulated) in the eclipse stage, 13 lncRNAs (13 up regulated) in the acute stage, 10 lncRNAs (nine up upregulated and one down regulated) in the post-seroconversion p31 negative stage and seven lncRNAs (six up regulated and one down regulated) in the post-seroconversion p31 positive stage were deregulated when compared to samples from uninfected individuals. (*p < 0·05; **p < 0·01, ***p < 0·001, ****p < 0·0001 and ns = non-significant as calculated using One-Way ANOVA)

**Fig. 5:**
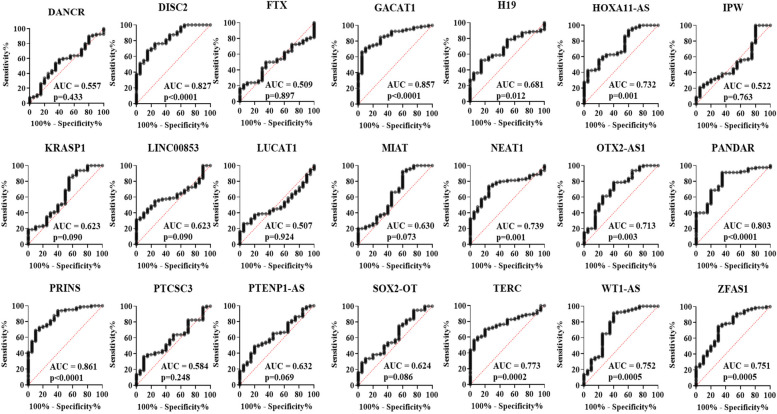
Receiver operating characteristic (ROC) curve analysis of differentially expressed lncRNAs in HIV-1 infected individuals vs. uninfected individuals in the validation stage

**Fig. 6:**
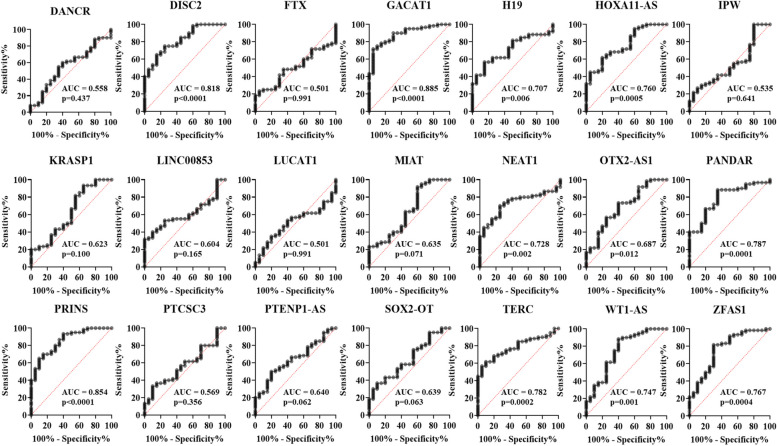
Receiver operating characteristic (ROC) curve analysis of differentially expressed lncRNAs in early HIV-1 infection vs. uninfected individuals in the validation stage

Since our goal was to identify potential biomarkers to detect HIV-1 in the early stage of infection, we focused on the 21 lncRNAs (Supplementary Table S1) that were differentially expressed in HIV-1 infected patients for further validation by qPCR. These lncRNAs were selected based on their fold change difference between the analyzed groups and their low Ct values, indicating that these lncRNAs might be abundant and easily detected in plasma. Next, the selected lncRNAs were validated in a larger sample size (80 plasma samples from HIV-1 infected individuals and 20 plasma samples from uninfected individuals) using quantitative RT-PCR. In this validation stage, comparison of the lncRNA expression profiles in plasma samples from HIV-1 infected individuals with the uninfected control population indicated that the expression of twelve lncRNAs were significantly increased and two lncRNAs were significantly decreased in the plasma from HIV-1 infected individuals (Fig. 2, Figure S3). In early HIV-1 infection, thirteen lncRNAs were significantly up regulated and two lncRNAs were significantly down regulated when compared to uninfected control population (Fig. 3, Figure S3). Likewise, two lncRNAs (1 down regulated and 1 upregulated) in the eclipse stage, thirteen lncRNAs were upregulated in the acute stage, ten lncRNAs (9 upregulated and 1 down regulated) in the post-seroconversion p31 negative stage, and seven lncRNAs (6 upregulated and 1 down regulated) in the post-seroconversion p31 positive stage were differentially expressed compared to the uninfected control population (Fig. 4, Figure S3). The diagnostic accuracy of these lncRNAs were evaluated using ROC analysis for detecting HIV-1 infection (Fig. 5), early HIV-1 infection (Fig. 6), eclipse stage, acute stage, post-seroconversion p31 negative stage, and post-seroconversion p31 positive stage (Supplementary Figure S4 -7). Results from the validation phase for DISC2, GACAT1, H19, HOXA11-AS, NEAT1, OTX2-AS1, PANDAR, PRINS, TERC, WT1-AS and ZFAS1 indicated that these lncRNAs were significantly modulated and were able to discriminate individuals with HIV-1 infection from normal uninfected control population. These lncRNAs had an AUC value of 0.827,0.857, 0.681, 0.732, 0.739, 0.713, 0.803, 0.861, 0.773, 0.752 and 0.751 respectively (Fig. 5). The results from the validation phase for DISC2, GACAT1, H19, HOA11-AS, NEAT1, OTX2-AS1, PANDAR, PRINS, TERC, WT1-AS and ZFAS1 indicated that these lncRNAs could significantly distinguish early HIV-1 infection from uninfected control population and the AUC value for these lncRNAs was 0.818, 0.885, 0.707, 0.760, 0.728, 0.687, 0.787, 0.854, 0.782, 0.747 and 0.767 respectively (Fig. 6). Results indicated that several lncRNAs were able to discriminate between eclipse stage, acute stage, post-seroconversion p31 negative stage, and post-seroconversion p31 positive stage of HIV-1 infection from the uninfected control population (Supplementary Figure S4-S7). For discrimination between eclipse stage of HIV-1 infection and uninfected control population, GACAT1 had the highest level of AUC of 0.847 among all other lncRNAs (Supplementary Figure S4). But for the acute stage of infection lncRNA GACAT1and PRINS exhibited an increased AUC (0.910) and could be differentiated from the uninfected control population (Supplementary Figure S5). PANDAR and PRINS had an AUC value of 0.897 (95% CI 0.803–0.991) and 0.902 (95%CI 0.809–0.995) respectively and could discriminate between post-seroconversion p31 negative stage of HIV-1 infection from the uninfected control population (Supplementary Figure S6). LncRNAs DISC2, PANDAR and PRINS had an AUC value of 0.855 (95% CI 0.740–0.970), 0.853 (95% CI 0.735–0.970) and 0.883 (95% CI 0.774–0.991) respectively could discriminate between post-seroconversion p31 positive stages of HIV-1 infection from normal uninfected control population (Supplementary Figure S7).

**Fig. 7:**
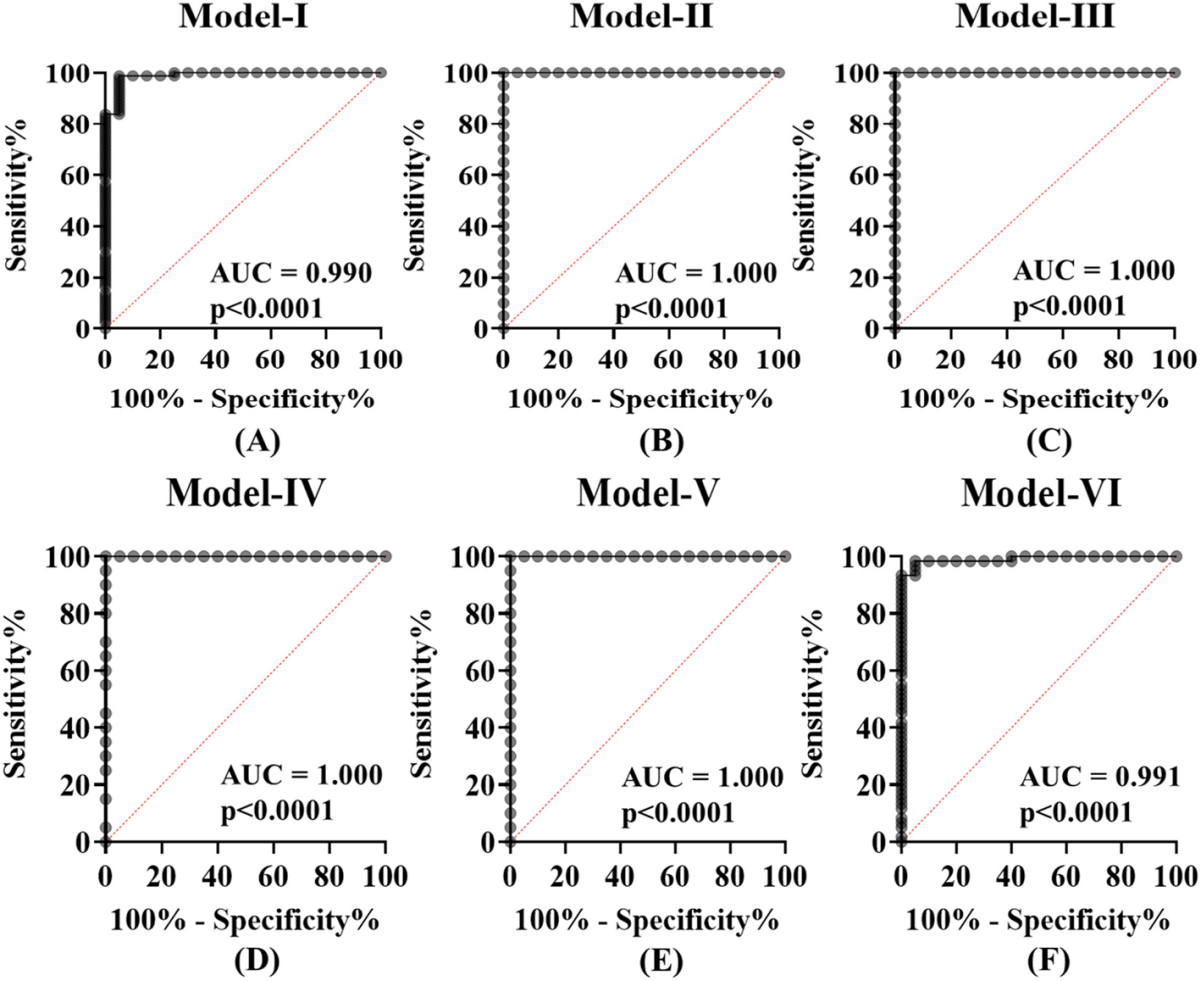
Receiver operating characteristic (ROC) curve analysis of lncRNA panels in the validation stage. **A** Model-I (DISC2, H19, IPW, KRASP1, NEAT1, PRINS, WT1-AS and ZFAS1) in HIV-1 infected individuals vs. uninfected individuals, (**B**) Model-II (DISC2, GACAT1, H19 and NEAT1) in the eclipse stage of HIV-1 infection vs. uninfected individuals, (**C**) Model-III (FTX, GACAT1, H19 and NEAT1) in the acute stage of HIV-1 infection vs. uninfected individuals, (**D**) Model-IV (GACAT1, MIAT, PANDAR and ZFAS1) in the post-seroconversion p31 negative stage of HIV-1 infection vs. uninfected individuals, (**E**) Model-V (IPW, KRASP1, NEAT1, OTX2-AS1, PRINS and WT1-AS) in the post-seroconversion p31 positive stage of HIV-1 infection vs. uninfected individuals, and (**F**) Model-VI (H19, NEAT1, ZFAS1, DANCR, GACAT1 and PRINS) in the early stage of HIV-1 infection vs. uninfected individuals

A logistic regression analysis was used to determine the best combination of lncRNAs to detect HIV-1 infection. The results of the analysis indicated that a linear combination of expression levels of DISC2, H19, IPW, KRASP1, NEAT1, PRINS, WT1-AS and ZFAS1 produced the best model to detect all stages of HIV-1 infection. The predicted probability of detecting HIV-1 infection by P_model−I_ was calculated by logit (model-I) = 11.866–0.953*DISC2–1.010*H19 + 0.649*IPW + 0.388*KRASP1 + 0.880*NEAT1–1.220*PRINS – 0.728*WT1-AS + 2.318*ZFAS1. The eight lncRNA signature panel showed significant detection of all the stages of HIV-1 infection. Compared with the use of each lncRNA alone this panel increased the AUC value to 0.990 (95% CI, 0.972-1·00, *P* < 0·00001: Fig. 7; Table 2) with 98.75% sensitivity and 95% specificity. Likewise, all lncRNAs were included in the logistic regression analysis to differentiate eclipse stage vs. uninfected control population, acute stage vs. uninfected control population, post-seroconversion p31 negative stage vs. uninfected control population, post-seroconversion p31 positive stage vs. uninfected control population, and early HIV-1 infection vs. uninfected control population. The selected lncRNAs were grouped into panels designated as P_model−II_, P_model−III,_ P_model−IV,_ P_model−V_ and P_model−VI_ for eclipse, acute HIV-1, post-seroconversion p31 negative, post-seroconversion p31 positive and early HIV-1 detection (Table 2). The cut-off values of the diagnostic performances of these models were determined based on the maximum of Youden index (P_model−II_ ≥ 0.265 for eclipse stage vs. uninfected control, P_model−III_ ≥ 0·191 for acute vs. uninfected control population, P_model−IV_ ≥ 0·019 for post-seroconversion p31 negative stage vs. uninfected control population, P_model−V_ ≥ 0·286 for post-seroconversion p31 positive stage vs. uninfected control population, P_model−VI_ ≥ 0.902 for early HIV-1 vs. uninfected control population ). 100%, 100%, 100%, 100%, 98.33% sensitivity, and 100%, 100%, 100%, 100% and 95% specificity were demonstrated between eclipse stage, acute stage, post-seroconversion p31 negative stage, post-seroconversion p31 positive stage, and early HIV-1 respectively compared with uninfected control population (Table 2).

**Table 2:** LncRNA panels for diagnosis of HIV-1 in validation datasets

lncRNA panel	Targeted stage of Disease/Disease vs. Control	Model	Cut-off value	Sensitivity	95% CI Sensitivity	Specificity	95%CI Specificity
P_model−I_	HIV-1	Logit (HIV-1) = 11.866–0.953*DISC2–1.010*H19 + 0.649*IPW + 0.388*KRASP1 + 0.880*NEAT1–1.220*PRINS – 0.728*WT1-AS + 2.318*ZFAS1	≥ 0.02211	98.75%	93.25–99.94%	95%	76.39–99.74%
P_model−II_	Eclipse	Logit (Eclipse) = 45.882–14.442*DISC2–4.313*GACAT1–40.097*H19 + 30.077*NEAT1	≥ 0.2650	100%	83.89–100.0%	100%	83.89–100.0%
P_model−III_	Acute HIV-1	Logit (Acute) = 191.880–27.647*FTX − 21.206*GACAT1–35.038*H19 + 31.147*NEAT1 + 8.623*PRINS	≥ 0.1910	100%	83.89–100.0%	100%	83.89–100.0%
P_model−IV_	Post-seroconversion p31 negative	Logit (Post-seroconversion p31 negative) = 81.792–15.964*GACAT1 + 10.798*MIAT − 17.564*PANDAR + 15.986*ZFAS1	≥ 0.01945	100%	83.89–100.0%	100%	83.89–100.0%
P_model−V_	Post-seroconversion p31 positive	Logit (Post-seroconversion p31 positive) = 826.946 + 52.825*IPW + 18.150*KRASP1 + 38.680*NEAT1–70.064*OTX2-AS1–52.646*PRINS − 78.381*WT1-AS	≥ 0.2866	100%	83.89–100.0%	100%	83.89–100.0%
P_model−VI_	Early HIV-1	Logit (Early) = 6.276–0.919*H19 + 0.554*NEAT1 + 2.258*ZFAS1 + 0.471*DANCR − 0.713*GACAT1–0.830*PRINS	≥ 0.9025	98.33%	91.14–99.91%	95%	76.39–99.74%

**Fig. 8:**
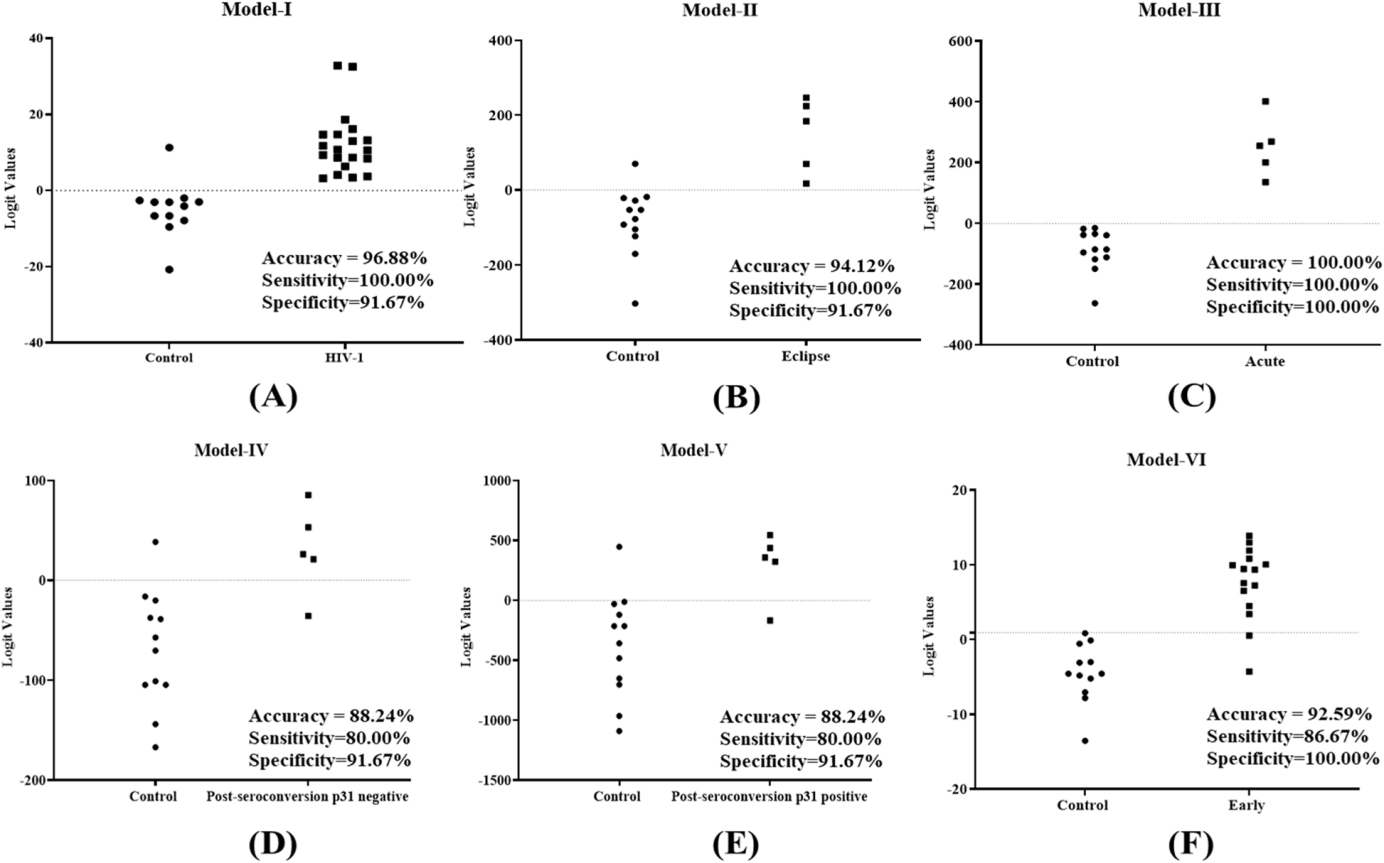
Logistic regression analysis- logit values from the panel test phase. The level of plasma lncRNAs from 20 HIV-1 positive individuals (eclipse stage *n* = 5, acute stage *n* = 5, post-seroconversion p31 negative stage *n* = 5 and post-seroconversion p31 positive stage *n* = 5) and 12 plasma samples from uninfected individuals were examined using real-time RT-PCR and normalized with two reference genes (RPLPO and RN7SK) as the control. Logit values were derived from regression analysis (**A**) Model-I for detection of HIV-1 infection. The results showed that HIV-1 infection was detected in samples from uninfected individuals with 96.88% accuracy and 100% sensitivity (**B**) Model-II for detection of eclipse stage of HIV-1 infection. The results showed that eclipse stage was detected from uninfected individuals with 94.12% accuracy and 100% sensitivity (**C**) Model-III for acute stage. The results showed that acute stage was detected from samples from uninfected individuals with 100.00% accuracy and 100% sensitivity (**D**) Model-IV for post-seroconversion p31 negative stage. The results showed that post-seroconversion p31 negative stage was detected from uninfected individuals with 88.24% accuracy and 80% sensitivity (**E**) Model-V for post-seroconversion p31 positive stage. The results showed that post-seroconversion p31 positive stage was detected from uninfected individuals with 88.24% accuracy and 80% sensitivity (**F**) Model-VI _for_ early HIV-1 infection. The results showed that early HIV-1 infection was detected from uninfected individuals with 92.59% accuracy and 86.67% sensitivity

Next, in the panel test phase, to validate the accuracy of the previously built diagnostic model, 20 plasma samples from HIV-1 infected patients and 12 uninfected individuals were tested. All these samples were independent samples unrelated to the two earlier test phases (discovery and validation phases). The sensitivity and specificity were calculated based on the models established in the validation stage. The results showed that, a signature composed of the eight lncRNAs (Model-I) correctly discriminated HIV-1 infection from uninfected control population with 100% sensitivity and 91.67% specificity (Fig. 8A). The results showed that Model-II could detect the eclipse stage from uninfected control population with an accuracy of 94.12%, Model-IV could detect the post-seroconversion p31 negative stage from uninfected control population with an accuracy of 88.24%, Model-V could detect the post seroconversion p31 positive stage from uninfected control population with an accuracy of 88.24% and Model-VI could detect early HIV-1 samples from uninfected control population with an accuracy of 92.59% respectively (Fig. 8B, D and E). In addition, our data suggest that the plasma lncRNA panel Model-II has excellent sensitivity and specificity as a biomarker for detecting acute HIV-1 infection (Fig. 8C).

**Fig. 9:**
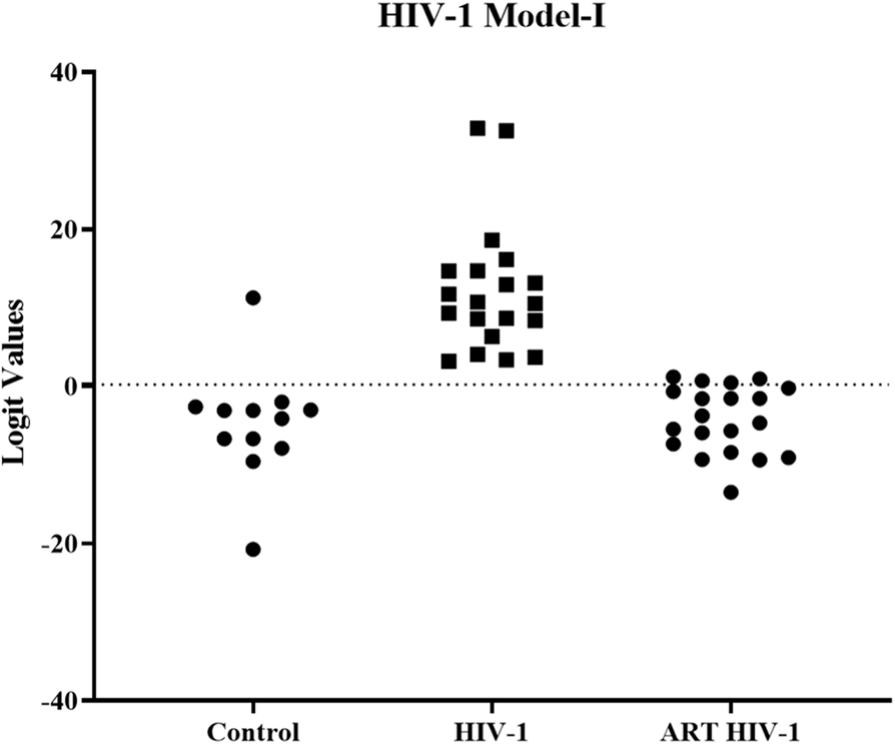
Logistic regression analysis- logit values for Model-I from the panel test phase samples. The level of plasma lncRNAs from 20 HIV-1 positive individuals, 20 ART-treated HIV-1 positive individuals and 12 plasma samples from uninfected individuals were examined as using real-time RT-PCR and normalized with two reference genes (RPLPO and RN7SK) as the control. Logit values were derived from Model-I. The values were measured in HIV-1 infected patients and in HIV-1 infected patients on ART treatment. The dotted line is representative of the cut-off values for samples from HIV-1 infected individuals compared to samples from healthy uninfected individuals

To identify the modulation of lncRNAS in response to ART in patients with HIV-1 during their treatment course, expression levels of the eight lncRNAs in the Model-I (11.866–0.953*DISC2–1.010*H19 + 0.649*IPW + 0.388*KRASP1 + 0.880*NEAT1–1.220*PRINS – 0.728*WT1-AS + 2.318*ZFAS1) were assessed. We measured the expression of eight lncRNAs in 20 plasma samples from ART treated HIV-1 infected patients using qRT-PCR. All the samples from HIV-1 infected patients on ART were obtained from patients who had received ART for at least 12 weeks (standard of care) and had undetectable (< 100 copies/ml) viral load after the treatment. The logit values of the eight lncRNA signature panel specific to HIV-1 infection when compared to uninfected control population were trending towards the uninfected control population (Fig. 9), suggesting that in HIV-1 infected patients on ART with undetectable levels of viral RNA, the differentially expressed lncRNAs in response to HIV-1 infection are restored to their normal expression levels (Supplementary Figure S8).

## Discussion

The World Health Organization reported that approximately 39 million people around the world were infected with HIV in 2023 and about 1.3 million new infections were documented in 2022. Thus, even after three decades of HIV discovery, AIDS is a serious health issue. Although, with the aggressive implementation of Highly active antiretroviral therapy and PrEP we are now capable of controlling the severity of disease progression and improving the lifespan of HIV infected individuals. Identifying individuals with acute HIV-1 infection is critical to prevent further HIV-1 transmission. Numerous studies have focused on miRNAs as potential biomarkers for detecting HIV-1 infection [35]. However very little is known about the utility of using lncRNAs to detect HIV-1 infection. In the context of HIV-1 infection, several lncRNAs have already been described to play a role during HIV-1 infection in vitro, either by being hijacked by HIV-1 to aid in replication or in the establishment of latency, or as part of the immunological response against the virus [30]. Here, we performed this study to identify potential lncRNAs that could serve as biomarkers for the detection of HIV-1 in their early stage of infection. With the increased use of antiretroviral treatment options, host biomarker-based detection tests may outperform traditional HIV-1 marker-based detection assays for detecting early HIV-1 infection. In our current study, we have included ART-treated samples and found that retrospectively altered lncRNAs converged toward normal levels, indicating treatment responsiveness for HIV-1 infection.

In this study, we employed a hypothesis-driven strategy to screen 84 lncRNA candidates that are among the most abundant and functionally relevant lncRNAs found in cells to date [42]. After the primary screen of lncRNA in the discovery phase, 21 lncRNAs were selected for subsequent validation phase. Through the validation process, 19 potential lncRNAs were identified that were differentially expressed in HIV-1 infection, eclipse stage, acute stage, post-seroconversion p31 negative stage, and post-seroconversion p31 positive stage and early HIV-1 stage compared with uninfected normal controls. All the lncRNAs had an AUC value less than 1·00, and less than 100% sensitivity and specificity. We have included all lncRNAs identified in our study in the multivariate analysis. A combined panel of lncRNAs significantly increased the accuracy of detection and improved the specificity and sensitivity of diagnosis compared to using single lncRNAs. Thus, the lncRNA-based test that we have designed employs a panel of lncRNAs instead of an individual lncRNA to detect eclipse stage, acute stage, post-seroconversion p31 negative stage, and post-seroconversion p31 positive stage, early HIV-1 stage, and differentiate between HIV-1 infection from uninfected control population. Because of the complex molecular events involved in HIV-1 infection, a single biomarker may not fully reflect the variance of the disease progression process or disease-state-specific expression, resulting in the limited efficacy of a single biomarker for HIV-1detection.

Most of the published work on HIV-1 detection has concentrated on viral markers [12, 43, 44]. Here, our research has focused on discovering host-based lncRNAs as biomarkers for early HIV-1 infection to improve detection and customize stage specific treatment. To enable prompt detection of acute HIV-1 infection and promote the rapid start of ART, accurate HIV testing is required. But it might be difficult to detect the acute infection stage because the symptoms can be ambiguous or undetectable [45]. The putative viral biomarkers suggested for identifying acute HIV-1 infection have significant limitations because roughly 3–5% of HIV-positive individuals will have a negative RNA test result during the window period of their HIV infection [43]. A crucial part of reducing HIV-1 transmission is now recognized as being the early detection of recently acquired infections [46]. Modeling results indicate that up to 50% of adult HIV-1 infections are acquired from patients with acute or early HIV-1 infection [47, 48]. Additionally, the comparatively low amount of HIV-1 p24 antigen during the early stages of HIV-1 infection may make its detection challenging. On the other hand, circulating lncRNAs that are detectable by qPCR and originate from cell-derived microvesicles, exosomes, apoptotic vesicle bodies, and other undiscovered routes may be used as possible indicators of HIV-1 infection. Circulating lncRNAs have been discovered in a few studies that may act as biomarkers for HIV-1 infection [23, 49, 50]. None of the earlier reports have evaluated the ability of circulating lncRNAs to detect both the eclipse stage and the acute/early stage of HIV-1 infection. Here, we have for the first time demonstrated the utility of lncRNA-based panels for the detection of HIV-1 infection in the presence or absence of viral markers. We have created a panel of lncRNAs (P_model−III_) for this investigation that has 100% sensitivity and specificity for detecting acute HIV-1 infections.

With the use of the lncRNAs in the panel, we created a model P_model−I_ [Logit (HIV-1) = 11.866–0.953*DISC2–1.010*H19 + 0.649*IPW + 0.388*KRASP1 + 0.880*NEAT1–1.220*PRINS – 0.728*WT1-AS + 2.318*ZFAS1] and established the cutoff value (≥ 0 022) for identifying HIV-1 infection. We have used the lncRNA panels to successfully detect HIV-1 infection during the panel test phase. We correctly identified 20 out of the 20 HIV-1 infected samples (100% sensitivity), and 11 out of 12 uninfected control samples (91.67% specificity) by using the lncRNA panel P_model−I_. Further, we have also evaluated the utility of the lncRNAs in P_model−I_ with patients infected with HIV-1 who underwent ART therapy; the results showed that the levels of DISC2, H19, IPW, KRASP1, NEAT1, PRINS, WT1-AS, and ZFAS1 were significantly lower and had reverted to near baseline levels, i.e., to those of uninfected individuals. Similar to our findings in the present study, Zhou et al., reported a restored in the level of lncRNAs with treatment [50]. As a result, in the future, following more extensive validation, we may be able to create a novel lncRNA panel and establish a cutoff value to identify HIV-1 infection and therapeutic responses based on host biomarkers.

In this study, plasma levels of DISC2, H19, KRASP1, PRINS, and WT1-AS were higher in patients with HIV-1 infection in the eclipse stage, acute stage, post-seroconversion p31 negative stage, and post-seroconversion p31 positive stage compared to uninfected control population, while levels of lncRNAs NEAT1 and ZFAS1 were lower. It was previously reported that NEAT1 was down regulated in HIV-1 infected individuals [33, 51]. As previously reported, we have found several additional circulating lncRNAs in people with HIV-1 infection in our study [23, 30, 52]. When distinguishing individuals with HIV-1 infection from uninfected individuals, the panel (P_model−I_) made up of eight lncRNAs performed significantly better than any single lncRNA. Panel P_model−I_ had 100% sensitivity and 91.67% specificity to identify HIV-1 infection during the validation and panel test phases of the study. Most importantly, P_model−I_ was able to identify the eclipse stage of infection when HIV-1 RNA in plasma was still undetectable or below the detection limit. Our findings highlight the potential application of P_model−I_ as a non-invasive biomarker to detect the eclipse stage of HIV-1 infection. Several lncRNAs in this study displayed pronounced aberrant expression patterns during HIV-1 infection, and these lncRNAs returned to normal expression levels following ART. Consistent with our findings in the present investigation, Zhaou et al. found a restoration in the level of lncRNAs with therapy [49, 50].

## Conclusions

In conclusion, our data show that the panels of circulating lncRNAs described in this report are effective at differentiating each stage of HIV-1 infection—including the eclipse, acute, post-seroconversion p31 negative, post-seroconversion p31 positive, and early stages of HIV-1 infection from uninfected control population. Our research has shown that HIV-1 infection can result in aberrant lncRNA expression, which reverts to normal levels by successful ART therapy. Only 84 particular lncRNA candidates, which are among the most numerous and functionally significant lncRNAs discovered in cells, were examined utilizing PCR Arrays across all samples in the discovery set [42]. But there are presently 127,802 human lncRNA transcripts reported in the lncpedia database (https://lncipedia.org/, accessed September 29, 2023), and there might be more lncRNAs that could be helpful indicators of HIV-1 infection. If additional significant lncRNAs are found in the future, they could be tested and the current panel could be updated to include those that showed enhanced sensitivity while maintaining high specificity for HIV-1 infection. Further the results of testing could be used to build a clinically applicable model for stratification of the early stages of HIV-1 infection, monitor disease progression and identify new targets for therapeutic intervention.

### Supplementary Information

**Supplementary Material 1.**

## Data Availability

No datasets were generated or analysed during the current study.

## Abbreviations

HIV: Human Immunodeficiency Virus
AHI: Acute HIV-1 Infection
PrEP: Pre-exposure Prophylaxis
ART: Anti-Retroviral Therapy
lncRNAs: Long Non-coding RNAs
DNA: Deoxyribonucleic Acid
RNA: Ribonucleic Acid
AIDS: Acquired Immunodeficiency Syndrome
CAD: Coronary Artery Disease
PCR: Polymerase Chain Reaction
AUC: Area Under the ROC Curve
ROC: Receiver Operating Characteristic Curve
DANCR: Differentiation Antagonizing Non-Protein Coding RNA
DISC2: Disrupted in Schizophrenia 2
FTX: FTX Transcript, XIST Regulator
GACAT1: Gastric Cancer Associated Transcript 1
H19: H19 Imprinted Maternally Expressed Transcript
HOXA11-AS: Homeobox A11 Antisense RNA
IPW: Imprinted in Prader-Willi Syndrome
KRASP1: KRAS Proto-Oncogene, GTPase Pseudogene 1
LINC00853: Long Intergenic Non-Protein Coding RNA 853
LUCAT1: Lung Cancer Associated Transcript 1
MIAT: Myocardial Infarction Associated Transcript
NEAT1: Nuclear Paraspeckle Assembly Transcript 1
OTX2-AS1: Orthodenticle Homeobox 2 Antisense RNA 1
PANDAR: Promoter of CDKN1A Antisense DNA Damage Activated RNA
PRINS: Psoriasis Associated Non-Protein Coding RNA Induced by Stress
PTCSC3: Papillary Thyroid Carcinoma Susceptibility Candidate 3
PTENP1-AS: Phosphatase and Tensin Homolog Pseudogene 1 Antisense RNA
SOX2-OT: SRY-Box Transcription Factor 2 Overlapping Transcript
TERC: Telomerase RNA Component
WT1-AS: WT1 Antisense RNA
ZFAS1: Zinc Finger NFX1-Type Containing 1 Antisense RNA 1
RN7SK: RNA Component Of 7SK Nuclear Ribonucleoprotein
RPLP0: Ribosomal Protein Lateral Stalk Subunit P0
miRNA: Micro RNA
p31: HIV-1 p31 Protein (Integrase)
