# Supplementary material for: Identification of a circulating long non-coding RNA signature panel in plasma as a novel biomarker for the detection of acute/early-stage HIV-1 infection
Source: Biomark Res. 2024 Jun 12;12:61. doi: 10.1186/s40364-024-00597-7 (PMC11167902; doi:10.1186/s40364-024-00597-7)
Supplement: Supplementary file 1 — Supplementary Material 1. [file 40364_2024_597_MOESM1_ESM.docx]

**Identification of a circulating long non-coding RNA signature panel in plasma as a novel biomarker for the detection of acute/early-stage HIV-1 infection**

Santanu Biswas, Namrata Nagarajan, Indira Hewlett *, and Krishnakumar Devadas *

**Affiliation**:

Laboratory of Molecular Virology, Division of Emerging and Transfusion Transmitted Diseases, Center for Biologics Evaluation and Research, Food and Drug Administration, 10903 New Hampshire Avenue, Silver Spring, MD 20993-0002, USA.

*Address correspondence and reprint requests to Dr. Krishnakumar Devadas and Dr. Indira Hewlett

E-mail address:

Krishnakumar.Devadas@fda.hhs.gov

[Indira.Hewlett@fda.hhs.gov](mailto:Indira.Hewlett@fda.hhs.gov)

**Figure S1:** Raw Cq values for the housekeeping genes (RPLPO and RN7SK) from the samples


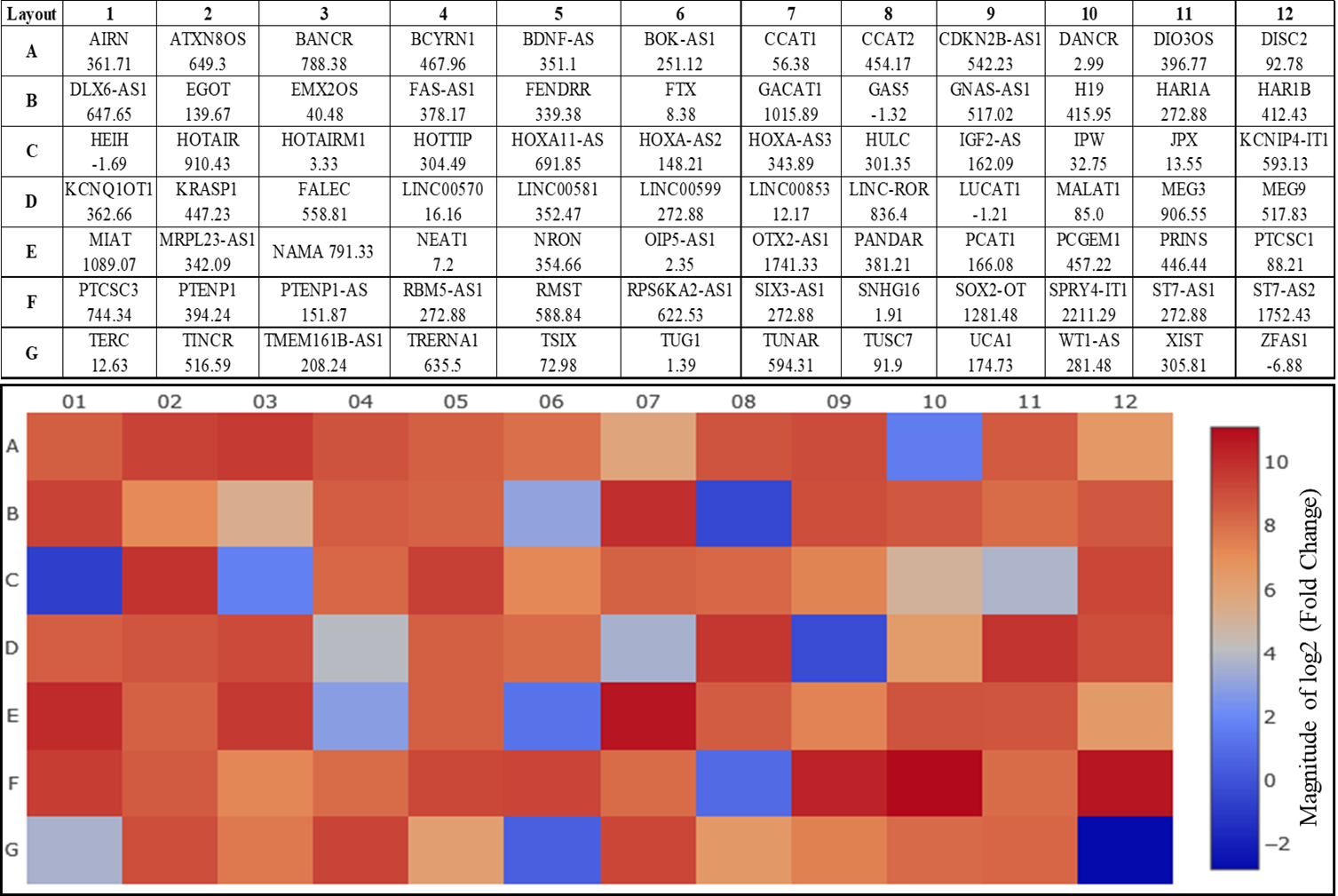
**(A)**

**(B)**

**Figure S2: Differential expression profiling of lncRNAs in HIV-1 infected patient population**. (A) Heat-map legend showing the lncRNA Profiler PCR array with all lncRNAs analyzed and their fold change in each box of the chart. (B) Heat-map profile showing the resulting PCR array dataset derived from the normalization of HIV-1 infected patients versus uninfected individuals. Red squares indicate upregulated lncRNAs, dark blue squares indicate downregulated lncRNAs, light blue squares indicate unchanged lncRNAs, and grey squares indicate technically unacceptable data. Technically unacceptable data was not considered in the analysis of our results.

**Table S1 List of selected lncRNAs from the PCR-array for further validation**

| **List of lncRNAs** | **Fold Change *** | | | | | | **p-value **** | | | | | |
| --- | --- | --- | --- | --- | --- | --- | --- | --- | --- | --- | --- | --- |
|  | **Eclipse** | **Acute ^#^** | **Post-seroconversion p31 negative** | **Post-seroconversion p31positive** | **Early HIV-1^⁋^** | **HIV-1** | **Eclipse** | **Acute ^#^** | **Post-seroconversion p31 negative** | **Post-seroconversion p31positive** | **Early HIV-1^⁋^** | **HIV-1** |
| DANCR | 1.18 | 4.54 | 4.04 | 2.73 | 3.08 | 2.99 | 0.346828 | 0.293666 | 0.188904 | 0.350201 | 0.305540 | 0.291193 |
| DISC2 | 11.98 | 123.21 | 243.79 | 123.43 | 83.63 | 92.78 | 0.762778 | 0.086127 | 0.163357 | 0.349099 | 0.288257 | 0.470893 |
| FTX | 0.64 | 13.26 | 11.50 | 26.63 | 5.51 | 8.38 | 0.842399 | 0.260566 | 0.146321 | 0.161263 | 0.324281 | 0.282169 |
| GACAT1 | 88.62 | 2320.95 | 2331.43 | 1207.03 | 954.15 | 1015.89 | 0.342975 | 0.035810 | 0.238751 | 0.312649 | 0.359051 | 0.333352 |
| H19 | 266.20 | 688.83 | 1140.14 | 128.07 | 638.39 | 415.95 | 0.786231 | 0.345079 | 0.133937 | 0.761745 | 0.390811 | 0.452113 |
| HOXA1-AS | 36.68 | 1458.48 | 1588.28 | 1293.89 | 551.00 | 691.85 | 0.885304 | 0.021904 | 0.206506 | 0.327832 | 0.298181 | 0.368955 |
| IPW | 1.17 | 12.29 | 88.83 | 392.69 | 13.27 | 32.75 | 0.408792 | 0.100408 | 0.013484 | 0.296855 | 0.204146 | 0.574333 |
| KRASP1 | 102.54 | 342.63 | 638.26 | 1234.53 | 309.16 | 447.23 | 0.133035 | 0.039616 | 0.128892 | 0.355106 | 0.169747 | 0.613189 |
| LINC00853 | 1.30 | 14.87 | 54.70 | 11.84 | 12.29 | 12.17 | 0.512179 | 0.576273 | 0.231161 | 0.386965 | 0.464091 | 0.453972 |
| LUCAT1 | 0.33 | 0.65 | 2.03 | 0.86 | 0.82 | 0.83 | 0.187166 | 0.262996 | 0.326678 | 0.604440 | 0.910035 | 0.833658 |
| MIAT | 347.38 | 342.63 | 6325.66 | 1404.17 | 992.94 | 1089.07 | 0.236408 | 0.039616 | 0.199271 | 0.137842 | 0.418001 | 0.429014 |
| NEAT1 | 2.00 | 15.23 | 9.75 | 6.56 | 7.44 | 7.20 | 0.273321 | 0.248329 | 0.146114 | 0.317521 | 0.286616 | 0.269449 |
| OTX2-AS1 | 190.18 | 3349.55 | 3511.20 | 2363.16 | 1558.33 | 1741.33 | 0.263742 | 0.019944 | 0.136518 | 0.332408 | 0.235936 | 0.320380 |
| PANDAR | 43.08 | 463.12 | 804.53 | 762.85 | 296.21 | 381.21 | 0.318120 | 0.036116 | 0.060730 | 0.222400 | 0.119009 | 0.200388 |
| PRINS | 43.23 | 2182.10 | 184.92 | 1270.34 | 305.22 | 446.44 | 0.548314 | 0.094380 | 0.416366 | 0.131080 | 0.302646 | 0.242371 |
| PTCSC3 | 89.72 | 856.91 | 2405.72 | 977.87 | 674.02 | 744.34 | 0.319612 | 0.058278 | 0.099681 | 0.306955 | 0.240867 | 0.256285 |
| PTENP1-AS | 21.35 | 341.98 | 144.51 | 308.74 | 117.34 | 151.87 | 0.474208 | 0.086927 | 0.291711 | 0.310554 | 0.204895 | 0.323854 |
| SOX2-OT | 31.38 | 338.15 | 8819.61 | 11398.05 | 578.86 | 1281.48 | 0.245690 | 0.093892 | 0.064487 | 0.240795 | 0.308181 | 0.479258 |
| TERC | 1.41 | 40.09 | 27.14 | 9.58 | 13.97 | 12.68 | 0.461224 | 0.226429 | 0.011335 | 0.076120 | 0.339784 | 0.368844 |
| WT1-AS | 33.46 | 190.18 | 474.41 | 1220.92 | 165.09 | 281.48 | 0.498303 | 0.627324 | 0.377452 | 0.218451 | 0.617874 | 0.528421 |
| ZFAS1 | 0.35 | 0.09 | 0.10 | 0.18 | 0.13 | 0.15 | 0.109764 | 0.018927 | 0.513514 | 0.050855 | 0.088270 | 0.041153 |

Some of the differentially expressed lncRNAs associated with HIV-1 infection were selected for further validation. The selection criteria were defined to meet the following requirements: 1) Ct values <38 either in samples from HIV-1 infected individuals or uninfected individuals to ensure stable detection; 2) relative fold change ≥2 (upregulation) or ≤0·5 (downregulation) between the HIV-1 infected individuals and uninfected individuals.

^*^Fold-Change is the normalized lncRNA expression in each test sample divided by the normalized lncRNA expression in the samples from uninfected individuals

^#^ Acute HIV-1group includes RNA+ and Ag+ samples

^⁋^ Early HIV-1 group includes Eclipse RNA+, Ag+ and post-seroconversion p31 negative samples

**p-value were calculated based on a student’s-test between two groups


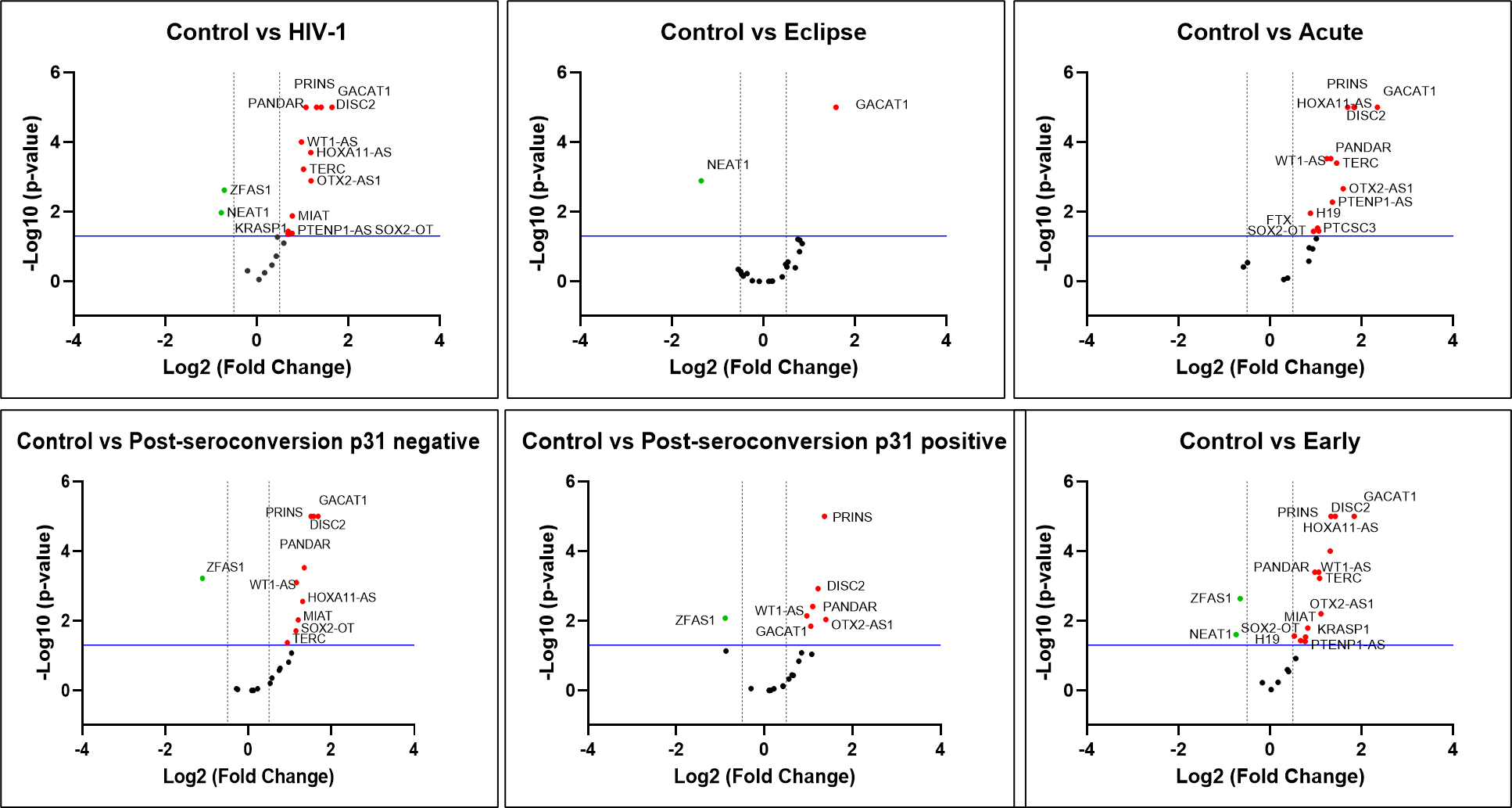


**Figure S3: Volcano plot of validation set.** Volcano plots of 21 lncRNA expression levels in the plasma of Control (n=20), Eclipse (n=20), Acute (n=20), post-seroconversion p31 negative (n=20) and post-seroconversion p31 positive (n=20) in the validation set. The x-axis is the estimated difference in expression measured in log2; vertical lines (black doted) refer to a 1.5-fold difference in expression between the two groups. LncRNAs highly expressed in HIV-1, eclipse, acute, post-seroconversion p31 negative, post-seroconversion p31 positive and early are on the right or the left, respectively. The y-axis is the significance of the difference measured in −log10 of the p-value; the horizontal blue line represents our cut-off for significance at p<0.05. Red dot and green dot are representing the significant upregulated and down regulated lncRNAs respectively.

**
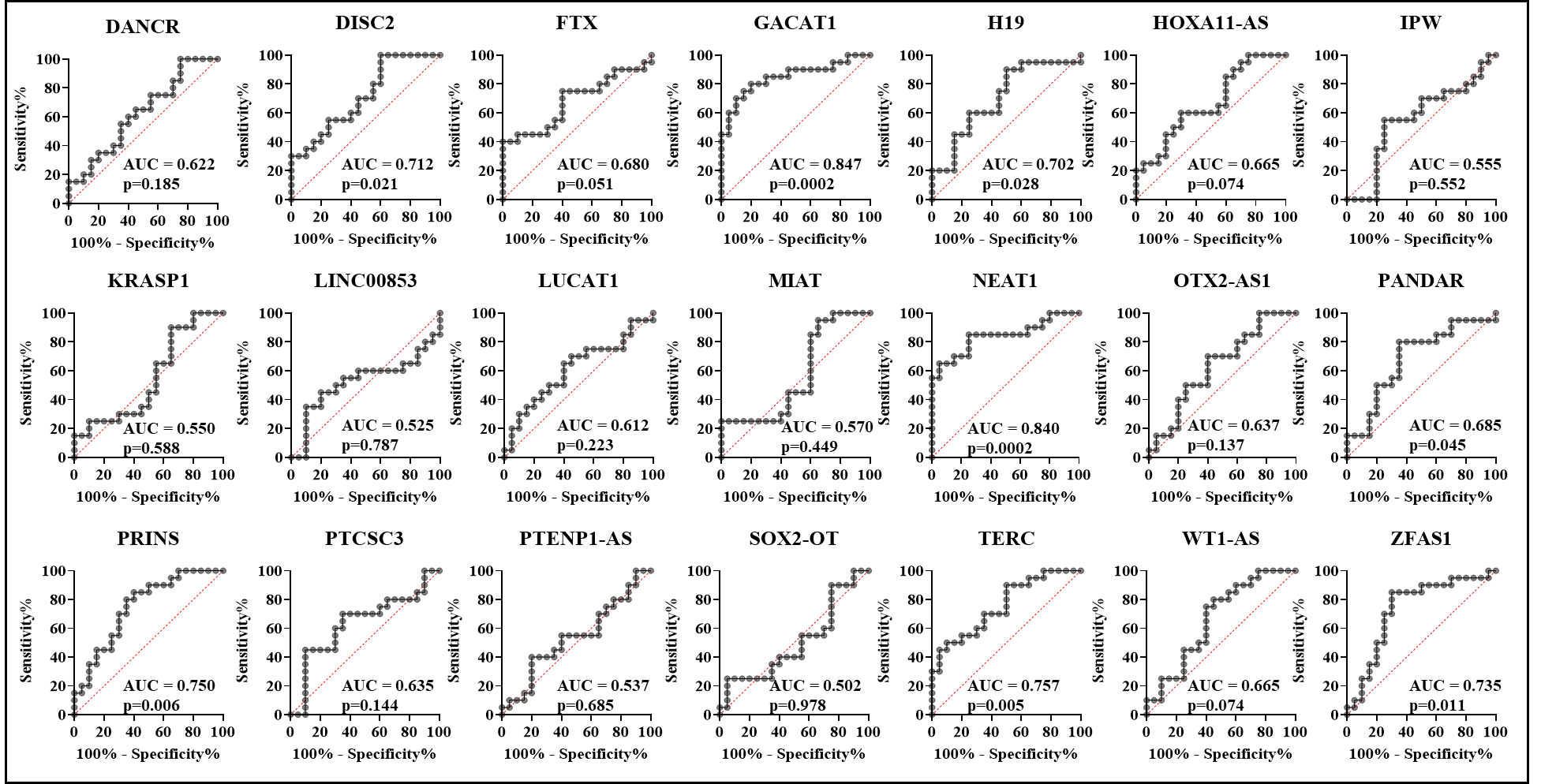
**

**Figure S4: Receiver operating characteristic (ROC) curve analysis for individual lncRNA in eclipse stage vs. uninfected individuals in the validation stage.**


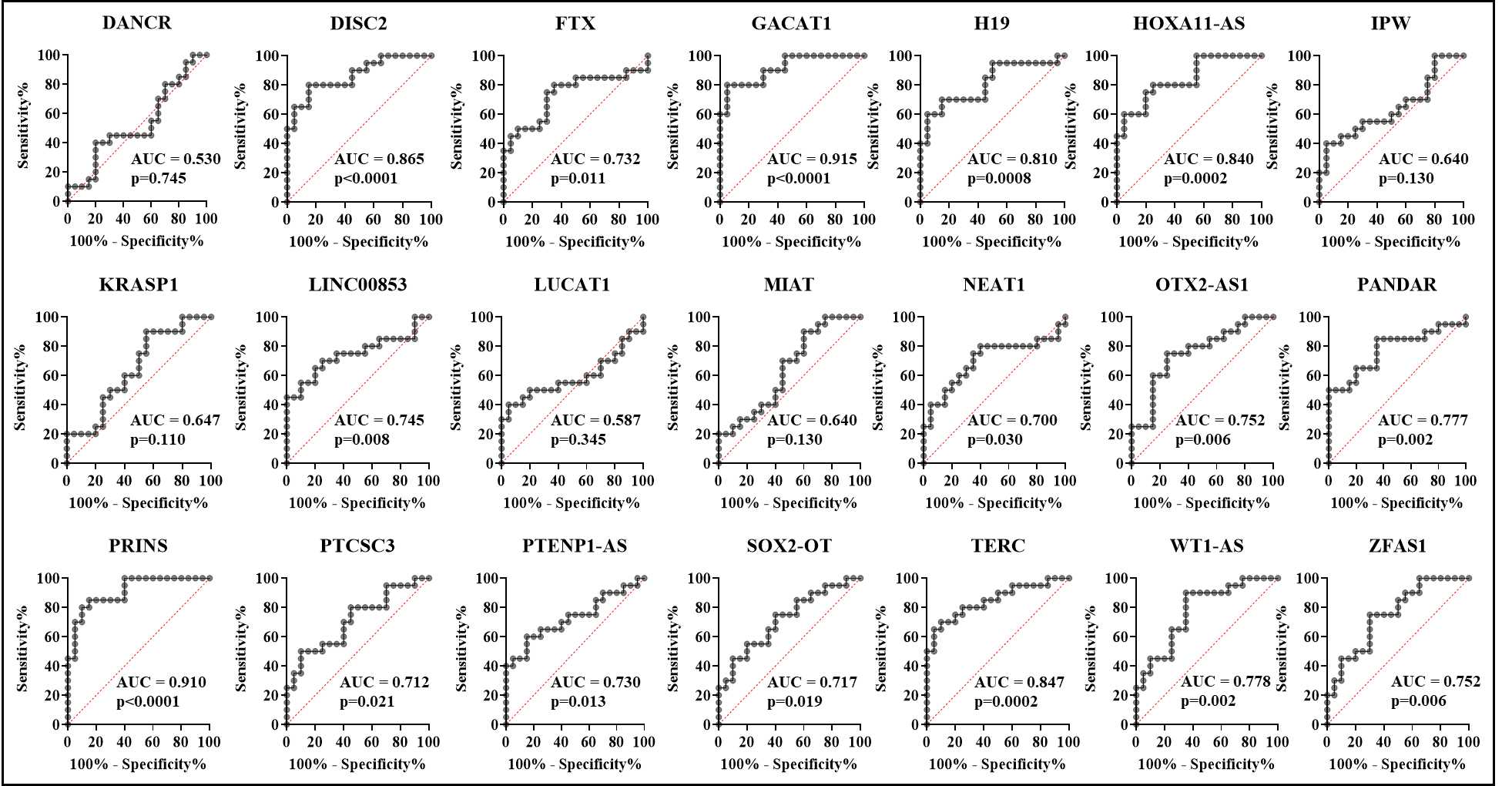


**Figure S5: Receiver operating characteristic (ROC) curve analysis for individual lncRNA in acute stage vs. uninfected individuals in the validation stage.**


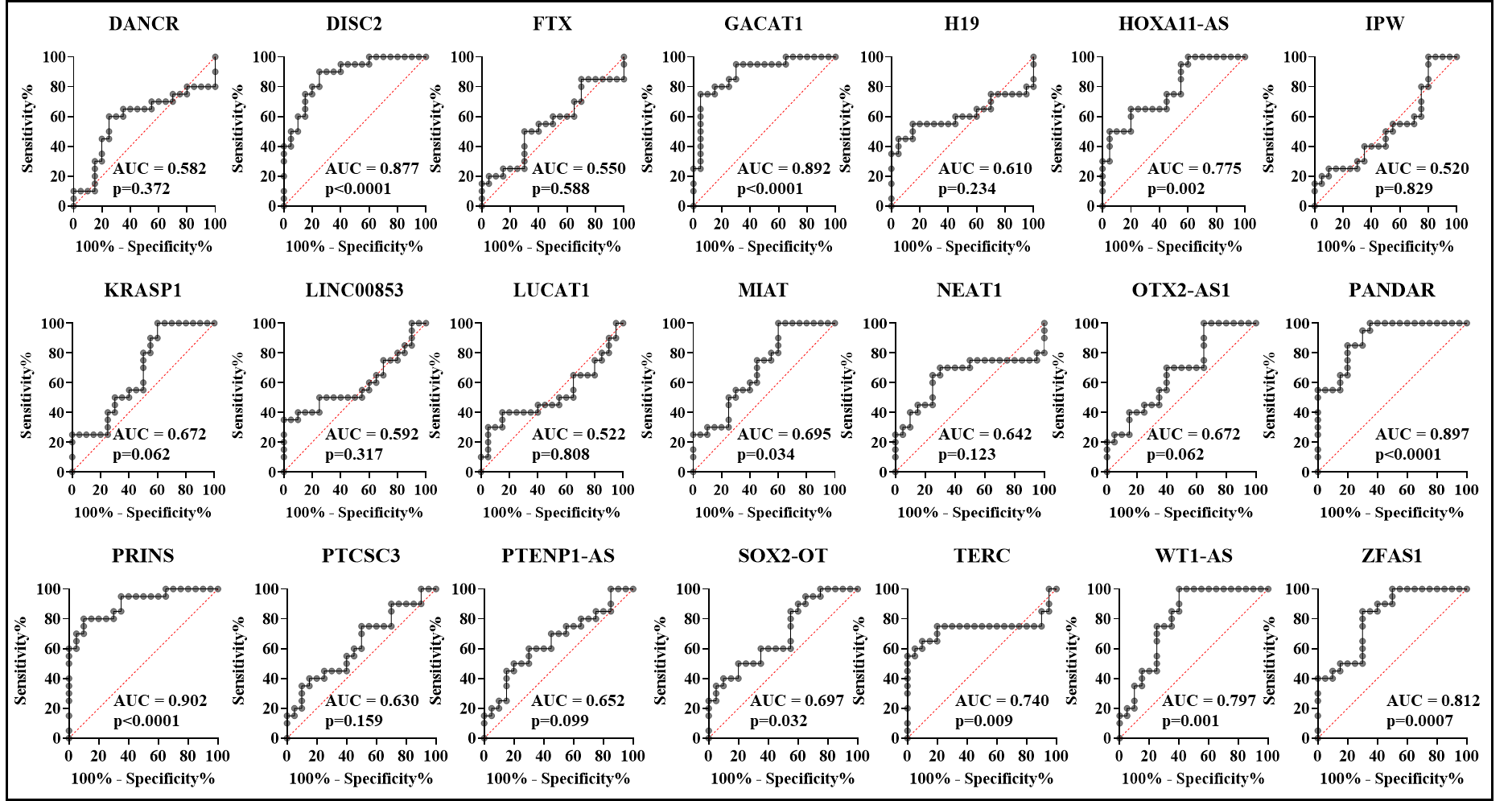


**Figure S6: Receiver operating characteristic (ROC) curve analysis for individual lncRNA in post-seroconversion p31 negative stage vs. uninfected individuals in the validation stage.**


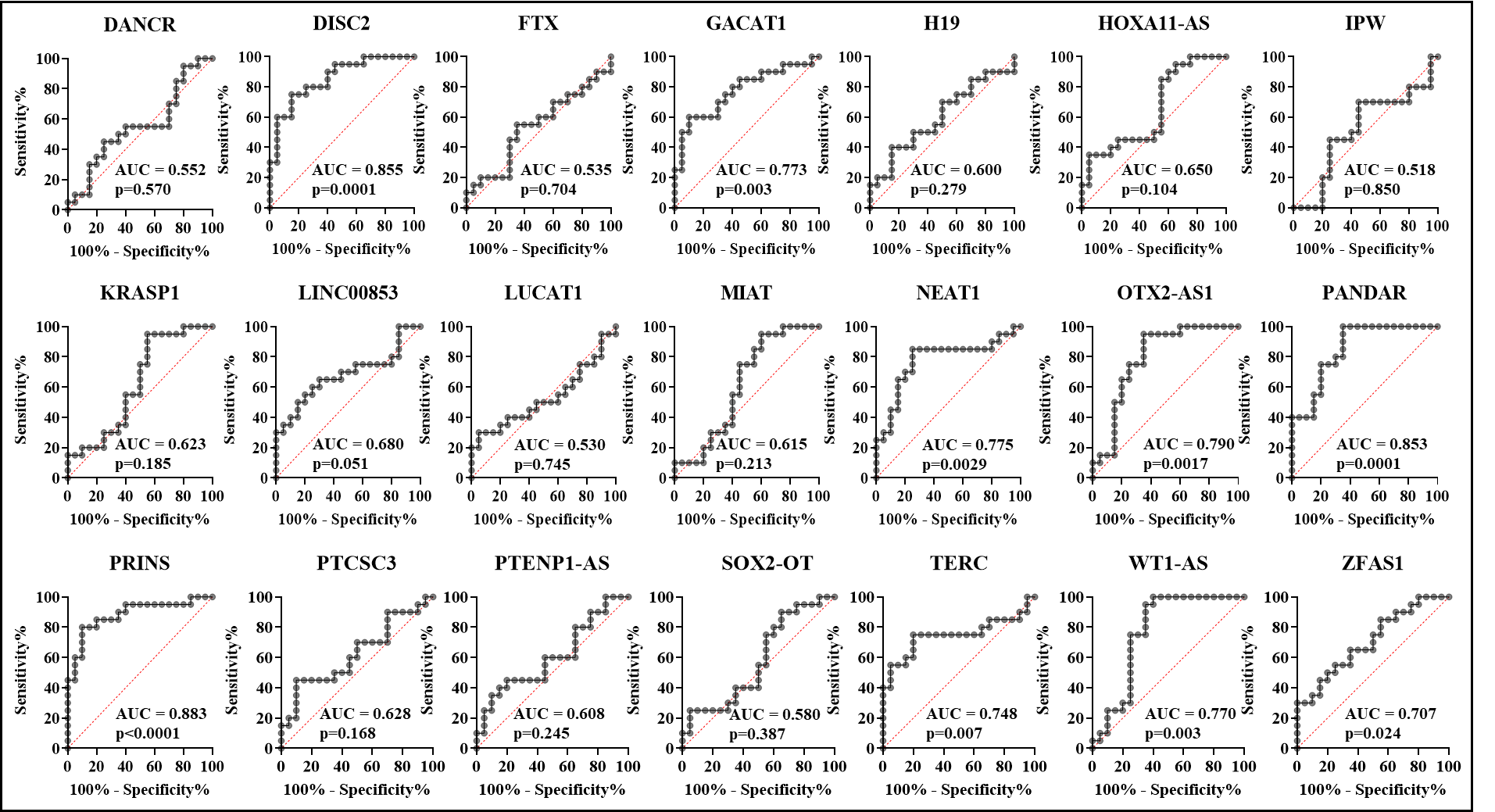


**Figure S7: Receiver operating characteristic (ROC) curve analysis for individual lncRNA in post-seroconversion p31 positive stage vs. uninfected individuals in the validation stage.**

**
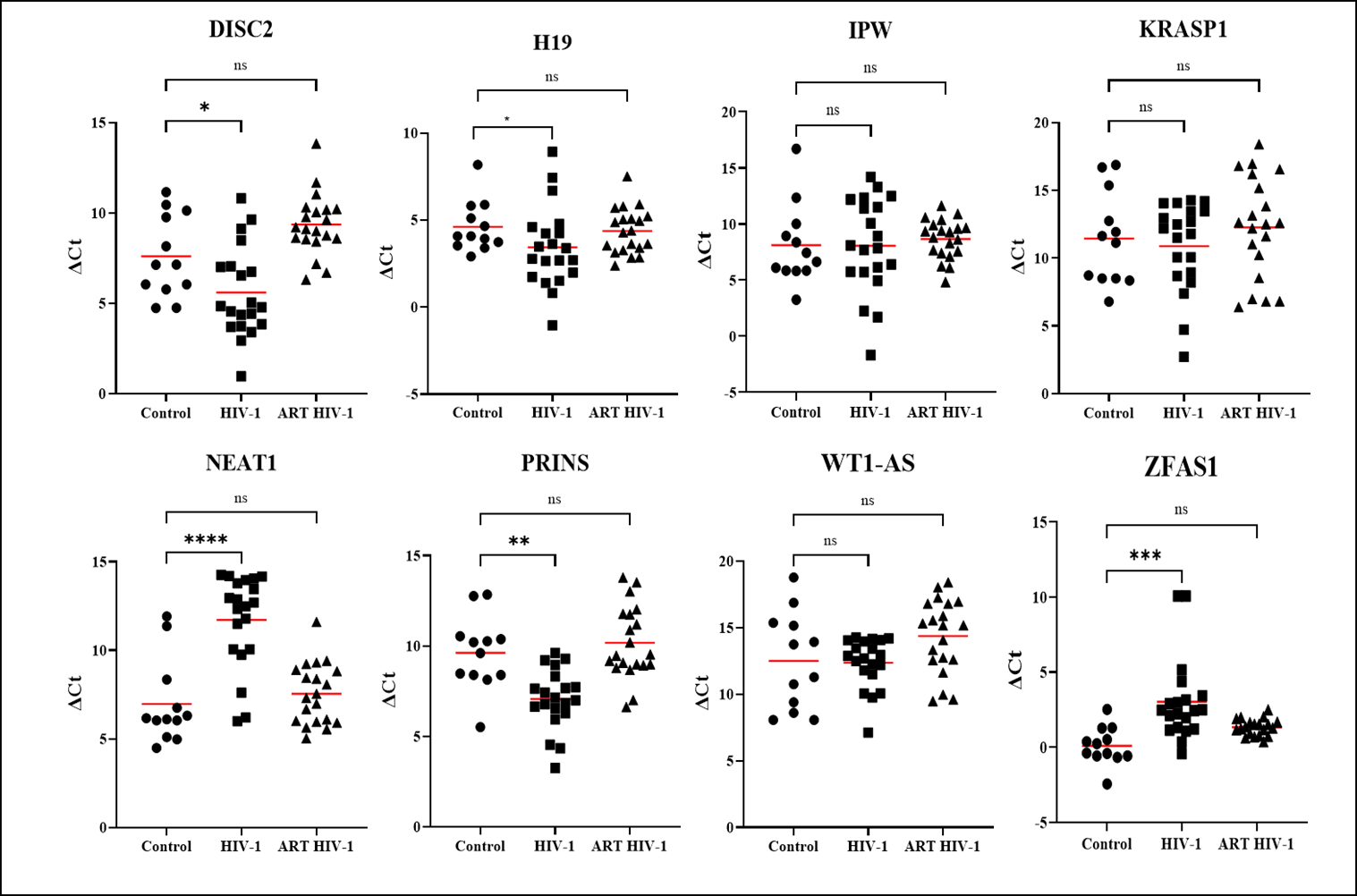
**

**Figure S8:** **Expression levels of eight plasma lncRNAs from P_model-I_ in the panel testing stage**. The level of plasma lncRNAs in 20 plasma samples from individuals with HIV-1infection, 20 plasma samples from HIV-1 infected individuals on ART and 12 plasma samples from uninfected healthy individuals were examined using real-time RT-PCR and normalized with two reference genes (RPLPO and RN7SK). Comparison of the lncRNA expression profiles in plasma samples from HIV-1 infected individuals on ART with the uninfected individuals indicated that the expression of eight lncRNAs significantly reverted towards the uninfected healthy individuals (*p < 0·05; **p < 0·01, ***p < 0·001, ****p < 0·0001 and ns=non-significant as calculated using One Way ANOVA test).
